# Phytochrome-Dependent Regulation of *ZFP6* and *ZFPH* Impacts Photomorphogenesis in *Arabidopsis thaliana*

**DOI:** 10.3389/fpls.2022.846262

**Published:** 2022-06-01

**Authors:** Keni Cota-Ruiz, Sookyung Oh, Beronda L. Montgomery

**Affiliations:** ^1^MSU DOE-Plant Research Laboratory, Michigan State University, East Lansing, MI, United States; ^2^Department of Biochemistry and Molecular Biology, Michigan State University, East Lansing, MI, United States; ^3^Department of Microbiology and Molecular Genetics, Michigan State University, East Lansing, MI, United States

**Keywords:** phytochrome, *ZFP6*, *ZFPH*, gibberellic acid, PIF, DELLA, far-red light

## Abstract

Phytochromes (phy) are key regulators of photomorphogenesis in plants. Among the different phys characterized in higher plants (i.e., phyA to phyE), phyA and phyB primarily regulate phenotypic responses in plants under far-red (FR) and red (R) conditions, respectively. Recent findings suggest that some zinc finger proteins (ZFPs) are involved in plant light-modulated morphogenesis. However, the interaction(s) between phyA, phyB and ZFP homologs potentially involved in photomorphogenesis, as well as their phenotypic and molecular effects in Arabidopsis seedlings exposed to R and FR light remain to be elucidated fully. Prior analyses with phytochrome chromophore deficient lines indicated that *ZFP6* expression is misregulated compared to levels in Col-0 wild type (WT). Here, we used plants with phytochrome chromophore or apoprotein (specifically phyA and phyB) deficiencies, lines with mutations in *ZFP6* and *ZFP6 HOMOLOG* (*ZFPH*) genes, and plants overexpressing *ZFP6* to examine regulatory interactions between phytochromes, ZFP6, and ZFPH. Our results indicate that phytochromes are required for downregulation of *ZFP6* and *ZFPH* and suggest a role for light-regulated control of *ZFP* levels in phytochrome-dependent photomorphogenesis. Conversely, *PHYB* is downregulated in *zfp6* mutants under R light. Analyses of a *zfp6zfph* double mutant confirmed disruption in photomorphogenic phenotypes, including the regulation of hypocotyl elongation in seedlings grown under FR light. In addition, *PIF3* and *PIF4* levels are transcriptionally regulated by ZFP6 and ZFPH in a gibberellic acid-dependent manner. *ZFP6* overexpression resulted in opposite phenotypic responses to those observed in the *zfp6* and *zfph* mutants grown in FR and R light, as well as a reduction in the rosette size of mature *ZFP6* OX plants relative to WT under white light. Based on these observations, we provide insight into how phy and ZFPs interact to regulate specific aspects of light-dependent processes in Arabidopsis.

## Introduction

Light controls multiple and critical processes throughout the plant life cycle. Aspects of plant growth and development regulated by light include seed germination, etiolation or de-etiolation behaviors in seedlings, responses to neighboring plants in competition for light, and the shift between vegetative and reproductive stages, among others (Fankhauser and Chory, 1997). These light-dependent growth and developmental processes are mediated by light perception by photoreceptors throughout the life cycle of plants, including phytochromes, cryptochromes, phototropins, and UVR8 (Legris et al., 2019). Phytochrome (phy) A (phyA) and phyB are the most extensively studied photoreceptors; they are the predominant phytochromes that control photomorphogenic responses in the presence of far-red (FR) and red (R) light, respectively (Li et al., 2011; Cheng et al., 2021; Kim et al., 2021). Encoded by genes in the nucleus, phy proteins are synthesized and the chromophore covalently attached in the cytoplasm; holophytochromes remain in the cytosol in their inactive form (Pr) if no activating light is present, or upon light-activated conversion to their active form (Pfr) are translocated into the nucleus (Kevei et al., 2007). In the nucleus, phytochromes control distinct classes of regulatory genes, including those encoding transcription factors.

Zinc finger proteins (ZFPs) are one class of transcription factor families that are widely distributed in plants. ZFPs participate in numerous biological processes, including flowering, light-mediated morphogenesis, disease suppression, and activation of defense mechanisms in response to abiotic stress (Feurtado et al., 2011; Noman et al., 2019; Xie et al., 2019). They are classified into nine families based on their conserved cysteine-histidine-amino acid motif, which coordinates with a zinc atom (Xie et al., 2019). The largest group comprises 176 C_2_H_2_-type ZFP proteins (Englbrecht et al., 2004). ZFPs have been shown to have DNA-binding activity in plants, indicating roles for these proteins in transcriptional regulation (Han et al., 2020). While one of the larger protein families, this group of regulatory proteins have been underexplored *in planta* (Fedotova et al., 2017). *ZFP6* and closely related *ZFP6 HOMOLOG* (*ZFPH*) are of particular interest in this current research given their identification as differentially regulated genes in prior transcriptomic analyses of phytochrome-deficient plant lines (Oh et al., 2013).

Prior experimental analyses demonstrated that *ZFP6* overexpression in 35S:*ZFP6* transgenic lines led to an increased number of trichomes on the sepals of flowers, in addition to ectopic trichome formation on carpels in Arabidopsis (Zhou et al., 2013). Of note, exogenous gibberellic acid (GA) application induced significantly higher *ZFP6* expression compared to untreated plants, indicating interaction between GA signaling and ZFP6 function (Zhou et al., 2013). The GA hormone is implicated in several plant development stages in Arabidopsis, including control of seed germination, promotion of stem elongation and leaf expansion, and the induction of flowering (Phillips, 1998). In addition to ZFP6, ZFP5 also aids in the GA pathway to induce trichome initiation on shoots in Arabidopsis (Zhou et al., 2011). Molecular-based approaches suggested that ZFP6 regulates *ZFP5* expression (Zhou et al., 2013) and ZFP5 in turn induces *GLABROUS INFLORESCENCE STEMS 1* (*GIS1*), *GLABROUS INFLORESCENCE STEMS 2* (*GIS2*), and *ZFP8* expression (Zhou et al., 2011). *GIS* genes also encode C_2_H_2_-type ZFPs. *ZFPH* was previously identified as *GIS3* and also was demonstrated to increase trichome density when overexpressed (Sun et al., 2015). *ZFPH/GIS3*, however, exerts its impact on trichomes independent of ZFP6 and ZFP5; yet, impacts *GIS1, GIS2*, and *ZFP8* similar to ZFP5 (Sun et al., 2015). Another *ZFP* family member, i.e., *ZFP3*, impacts seedling development, but through a distinct mechanism. Overexpression of *ZFP3* interfered with the ABA signaling pathway in Arabidopsis, rendering the seeds unable to germinate. Additionally, seedlings overexpressing *ZFP3* displayed shorter hypocotyls both in light and dark conditions (Joseph et al., 2014). Together, these results indicate multiple roles for *ZFP* homologs in plant growth and development, including some phenotypes that overlap with those controlled by light and phytochromes.

PIFs (Phytochrome-Interacting Factors) are phytochrome-dependent transcription factors that have been shown to physically interact with phytochromes and to activate organ-elongation genes and promote etiolation (Leivar and Monte, 2014), i.e., the dark-dependent development of seedlings with long stems and small, yellow-colored cotyledons. During de-etiolation, R light-dependent activation of phyB leads to degradation of PIFs and characteristic inhibition of stem elongation and promotion of leaf development and greening (Leivar and Monte, 2014). In addition to impacting PIFs, phyB inhibits the morphogenetic repressor COP1 (CONSTITUTIVE PHOTOMORPHOGENIC 1) in a light-dependent manner, restraining its ability to target the transcription factor ELONGATED HYPOCOTYL 5 (HY5) for proteasome-mediated degradation (Osterlund and Deng, 1998). Thus, HY5 accumulates in the light and promotes photomorphogenesis in plants (Osterlund et al., 2000; Shi et al., 2018). Conversely to its R-dependent movement into the nucleus, phyB remains in its inactive red-light absorbing form (Pr) in the cytoplasm under FR light conditions. When phyB remains in the cytosol in FR, PIF molecules are able to accumulate in the nucleus where they function to transcribe PIF target genes, including those that promote elongation (Ejaz et al., 2021). PIF proteins intersect with hormone-based regulation of growth as targets of the GA signaling pathway (Hernández-García et al., 2021). PIFs are targeted for inactivation by DELLA proteins, which are molecules that suppress growth (Kusnetsov et al., 2020). DELLAs restrain PIFs (PIF1, PIF3, PIF4, and PIF5) by targeting them for proteasome-mediated degradation in a light-independent manner (Li et al., 2016).

Given the prior associations of ZFP6 as a target of transcriptional regulation by phytochromes and GA regulation, as well as roles for both phytochromes and GA in light-dependent growth and development in Arabidopsis, we investigated light and phytochrome-dependent transcript accumulation for *ZFP6* and *ZFPH*, light and GA-dependent phenotypic and molecular responses of *zfp6* and *zfph* mutants, and the consequence of overexpressing the *ZFP6* gene on light-dependent plant growth and development. To gain specific insights into the crosstalk between phytochromes, PIFs, and ZFP6 during the regulation of growth, we assessed expression of select genes within the phytochrome, GA, and organ elongation pathways. Considering these observations collectively, we describe specific aspects of phytochrome-dependent processes that are mediated via ZFP6 and closely related ZFPH in Arabidopsis.

## Materials and Methods

### Plant Materials

Col-0 wild type (WT) ecotype of *Arabidopsis thaliana* (hereafter Arabidopsis) was obtained from the Arabidopsis Biological Resource Center (ABRC).^1^ An Arabidopsis *phyAphyB* (*PHYA*: *AT1G09570*; *PHYB*: *AT2G18790*) double mutant line was previously constructed and described (Mayfield et al., 2007; Ruckle et al., 2007). *zfp6* (SALK_200865; *AT1G68360*) and *zfph* (SALK_043793; *AT1G68360*) mutant lines were also obtained from ABRC, and the *zfp6zfph* double mutant was isolated from a genetic cross between the two single mutants. The production of transgenic BVR lines was previously described (Montgomery et al., 1999; Warnasooriya and Montgomery, 2009).

For *ZFP6* overexpression lines, *ZFP6* cDNA was amplified from a cDNA clone for *ZFP6* (U13157) from ABRC using forward primer 5′-ATGGCGACTGAAACATCTTCTT-3′ and reverse primer 5′-TCATGGCCCAAGGCTTAAAT-3′ and recombined into the pCR™8/GW/TOPO™ vector using a TA Cloning Kit according to manufacturer’s instructions (Thermo Fisher Scientific, Waltham, MA, United States). Insertion of the full-length *ZFP6* cDNA fragment into the vector was confirmed via *Eco*RI digestion and validated by DNA sequencing. The recombinant vector was cloned into One Shot™ TOP10 *E. coli* cells (Thermo Fisher Scientific, Waltham, MA, United States) following the manufacturer’s directions. Full-length *ZFP6* cDNA was recombined into the 35S promoter-containing pEarlyGate 100 vector using LR Clonase II enzyme according to manufacturer’s instructions (Thermo Fisher Scientific, Waltham, MA, United States) to generate the 35S:ZFP6 construct, which was introduced into GV3101 Agrobacterium to transform Col-0 WT plants via a standard floral-dip transformation protocol (Clough and Bent, 1998). *ZFP6* overexpression was confirmed by RT-PCR as described below.

### Plant Growth and Light Sources

Arabidopsis seeds were sterilized with 2.88% (v/v) sodium hypochlorite including 0.025% (v/v) SDS for 15 min. Chlorine was removed by rinsing seeds with sterilized ddH_2_O five times. Then, the seeds were planted on 0.5 × Murashige and Skoog (MS) medium (Caisson Laboratories, Smithfield, UT, United States) containing 0.9% (w/v) Phytoblend (Caisson Laboratories) and 1% (w/v) sucrose (Thermo Fisher Scientific, Waltham, MA, United States). Seeds were stratified on agar plates at 4°C for 4 days in darkness and then replicate plates were incubated in white (W), far-red (FR) and red (R) light for 7 days at 22°C. Additional treatments included germination and/or growth of seedlings on plates with 10 μM GA and GA biosynthesis inhibitor paclobutrazol (PAC) at a concentration of 100 nM. For W light, a Percival chamber model no. CU36LA irradiating light at 110 μmol m^–2^ s^–1^ was used; for the rest of the tested lights (see below), Percival LED chambers (model E30LED; Percival, Perry, IA, United States) were employed. For continuous FR (FR; λmax ∼735 nm) light, the light was emitted at 5 μmol m^–2^ s^–1^; for R conditions (λmax ∼670 nm), the fluence rate was ∼25 to 50 μmol m^–2^ s^–1^; and for blue (B) conditions (λmax ∼ 470 nm), the fluence rate was ∼50 μmol m^–2^ s^–1^.

### Phenotypic Analyses

Hypocotyl and root lengths of 7-days-old *zfp6, zfph*, and *zfp6zfph* mutant seedlings were measured using the ruler tool in Photoshop 2021 or using Image J. Similar measurements were performed on single copy, homozygous lines overexpressing *ZFP6* grown for 7 days in MS media containing 1% sucrose and 0.7% agar, pH 5.7, under FR (5 μmol m^–2^ s^–1^), R (50 μmol m^–2^ s^–1^), and blue (50 μmol m^–2^ s^–1^) lights at 22°C. To document additional phenotype characteristics of mature lines overexpressing *ZFP6*, Col-0 WT and 35S:*ZFP6* overexpression lines were grown in soil for 21 days at 22°C in W at ∼125 μmol m^–2^ s^–1^ under a 16 h light/8 h dark cycle. Plants were photographed to evaluate rosette architecture and trichome formation.

### RNA Extraction and RT-PCR

Seven-day-old seedlings incubated in R and FR light at 22°C were harvested in green light conditions, while those grown in W light were harvested under room light. Collected seedlings were immediately submerged in liquid nitrogen and stored at –70°C. Total RNA was extracted using the E.Z.N.A. Plant RNA Kit (Omega Bio-Tek, Norcross, GA, United States), following the manufacturer’s instructions and including the DNase I digestion protocol. An additional DNA digestion was performed using DNase I RNase-free (Thermo Fisher Scientific, Waltham, MA, United States) using 1 unit per microgram of RNA. Total RNA (500 ng) was reverse-transcribed using a qScript cDNA SuperMix kit (Quantabio, Beverly, MA, United States). Real-time quantitative PCR (qPCR) was performed on an ABI 7500 Fast Real-Time PCR System (Applied Biosystems, Foster City, CA, United States). The primers (at a final concentration of 200–500 nm) and cycling conditions are specified in Supplementary Table 1. A melting curve protocol was performed at the end of the PCR starting at 60°C with increments of 0.5°C/20 s. Three biological replicates along with three technical replicates were used. The *UBC21* gene was used for normalizing purposes and gene expression analyses were conducted using the 2^–Δ*CT*^ method. To confirm overexpression of *ZFP6*, standard RT-PCR was performed with *UBC21* as the internal control using primers (final primer concentration 400 nm) and cycling conditions indicated in Supplementary Table 1.

### *In silico* Promoter Analyses

Analyses of the *ZFP6* and *ZFPH* promoter regions were performed using the PlantCare database^2^ to identify conserved *cis*-elements potentially involved in gene regulation. Approximately 1,000 nucleotides upstream of the start codon of each gene were analyzed to search for transcription start (TS) sites using the neural network promoter prediction with a minimum promoter score of 0.9^3^ and for predicted transcription factor binding sites using the PlantCare database. In parallel, the TF2Network database (Kulkarni et al., 2018)^4^ was used to investigate potential light- and/or phytochrome-dependent regulators for *ZFP6* and *ZFPH*.

### Statistical Analysis

ANOVA analysis was performed to examine significant differences in the means. A normal distribution of the data was evaluated by the Kolmogorov-Smirnov test. Data that did not follow a normal distribution were transformed using the Box-Cox algorithm. The Fisher test at *p* ≤ 0.05 was conducted to evaluate significant differences among population means. All statistical analyses and graphs were generated on OriginPro 2018.

## Results

### Phytochrome A and Phytochrome B Negatively Regulate *ZFP6* and *ZFPH* Transcript Levels

**FIGURE 1:**
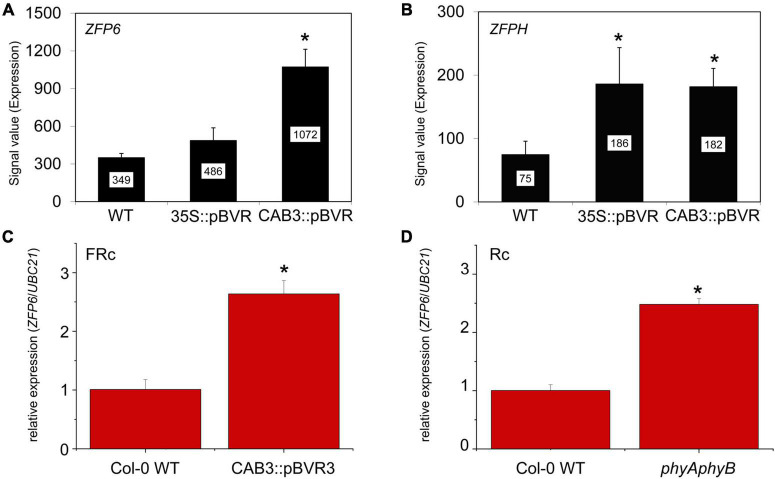
Relative expression of *ZFP6* homologs in Arabidopsis seedlings. Expression levels (signal value) of **(A)** *ZFP6* and **(B)** *ZFPH* in wild-type (WT), 35S:pBVR, and CAB3:pBVR seedlings grown under continuous far-red (FRc) from published data set (Oh et al., 2013) are shown (± SD, *n* = 3). Signal value indicates signal intensity on the ATH1 array as calculated by Affymetrix Microarray Suite (MAS). **(C)** *ZFP6* relative expression in CAB3:pBVR3 line (Warnasooriya and Montgomery, 2009) in FRc light compared to WT. **(D)** *ZFP6* relative expression in *phyAphyB* mutant in continuous red (Rc) light conditions compared to WT. **(C,D)** Seedlings were stratified at 4°C for 4 days in MS plates with 1% sucrose and then incubated in FRc or Rc light for 7 days. Gene expression data were obtained following the 2^–Δ*CT*^ method using *UBC21* as the reference gene. Means ± SD were calculated from at least three biological replicates. *p ≤ 0.05, relative to WT.

Mining of previous transcriptomic data indicated that *ZFP6* and *ZFPH* were differentially regulated in phytochrome chromophore-deficient transgenic *BVR* lines grown in FR light conditions (Oh et al., 2013). BVR (biliverdin IX reductase) inactivates the tetrapyrrole precursors required for synthesis of the phytochrome chromophore, phytochromobilin; thus, BVR induces a chromophore deficiency in transgenic plants (Montgomery et al., 1999). The mRNA levels for both *ZFP6* and *ZFPH* were significantly higher in FR-grown CAB3:pBVR lines that lack the accumulation of photoactive phytochromes in mesophyll cells of leaves (Figures 1A,B; Oh et al., 2013). To confirm this finding for *ZFP6*, we assessed its expression by quantitative, real-time PCR (qRT-PCR) analysis. *ZFP6* mRNA levels were ∼2.6-fold higher in a CAB3:pBVR line than in Col-0 WT grown in FR light (Figure 1C). As the lack of phytochrome chromophore results in a lack of all holophytochromes, we used a *phyAphyB* mutant lacking the two predominant phytochromes to confirm that it was the lack of phytochromes in the BVR line which directly contributed to a disruption in transcript accumulation for *ZFP* homologs. Consistent with the phenotype for chromophore-deficient *BVR*-expressing plants, *ZFP6* transcripts levels also were increased ∼2.5 fold in a *phyAphyB* T-DNA mutant line (Figure 1D), compared to Col-0 WT. These findings suggest that phyA and phyB are the primary phytochromes required to downregulate *ZFP6*.

### *ZFP6* and *ZFPH* Are Expressed in Different Tissues and in Response to Distinct Light Conditions

**FIGURE 2:**
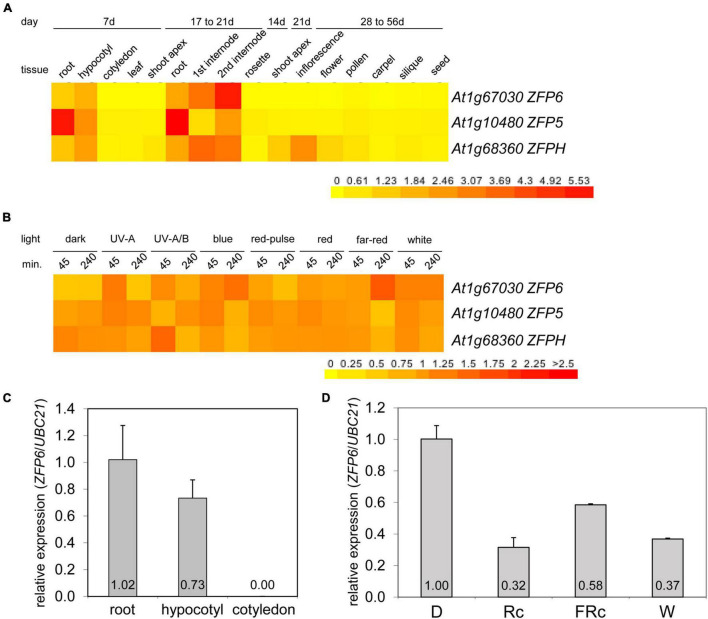
Expression of *ZFP6, ZFP5*, and *ZFPH* in different tissues and light conditions. Heat map showing the expression of *ZFP6, ZFP5*, and *ZFPH* in **(A)** different tissues or **(B)** different light conditions for Arabidopsis. For heat map, mean-normalized values of Col-0 WT from AtGenExpress expression library and BAR Heatmapper Plus (bar.utoronto.ca) were used. For light experiments in panel **(B)**, aerial parts (hypocotyl and cotyledons) of 4-days-old Col-0 WT seedling grown on MS medium were treated with different light for either 45 or 240 min. **(C)** qRT-PCR analysis of *ZFP6* expression in Col-0 WT root, hypocotyl, or cotyledon tissues from seedlings grown on MS medium containing 1% sucrose and 0.7% Phytoblend agar at 22°C for 7 days under white light (W) at 100 μmol m^–2^ s^–1^. **(D)** *ZFP6* expression analyses in Col-0 WT grown as mentioned in **(C)** but under dark, continuous red (Rc; 50 μmol m^–2^ s^–1^), continuous far-red (FRc; 5 μmol m^–2^ s^–1^), or W (100 μmol m^–2^ s^–1^) light conditions. Relative *ZFP6* expression level compared with *UBC21* is shown (± SD, *n* = 3).

Given the role of phys in regulating *ZFP6* and *ZFPH*, we examined the expression of these genes and the closely related *ZFP5* in different tissues and light conditions utilizing public microarray data for Col-0 WT from AtGenExpress^5^ (Figures 2A,B). We chose to examine expression of *ZFP5* in parallel given that it is regulated by ZFP6 (Zhou et al., 2013), shares regulation of similar GA-dependent phenotypic responses as *ZFP6* (Zhou et al., 2011), and controls expression of some of the same genes as ZFPH (Sun et al., 2015). *ZFP5* shared some overlap with *ZFP6* and *ZFPH* in terms of tissues in which it was expressed, including roots and hypocotyls; yet, *ZFP5* was expressed to relatively higher levels in roots than either *ZFP6* or *ZFPH* (Figure 2A). *ZFP6* and *ZFPH* are highly expressed in roots, hypocotyls, and internodes, with *ZFPH* also exhibiting some expression in the shoot apex and inflorescence tissues (Figure 2A). We focused our subsequent analyses on the most closely related *ZFP6* and *ZFPH*.

Using qRT-PCR, we confirmed the differential accumulation of *ZFP6* mRNA in roots and hypocotyls, but not in cotyledons (Figure 2C). Additionally, *ZFP6* is upregulated by light, with significant upregulation after 4 h of FR (10 μmol m^–2^ s^–1^) exposure according to public microarray data (Figure 2B). By comparison, *ZFPH* exhibits more moderate light-dependent changes in expression, with UV-A/B having the most significant impact. Given the association of multiple wavelengths of light that are correlated with phytochrome activity having a greater impact on *ZFP6* induction, we documented that *ZFP6* expression was 3. 1-, 1. 72-, and 2.7-fold downregulated in Col-0 WT exposed to continuous R, FR, and W light conditions, respectively, compared to Col-0 WT grown in dark (Figure 2D).

### ZFP6 and ZFPH-Deficient Lines Exhibit Defects in Light-Dependent Phenotypes

**FIGURE 3:**
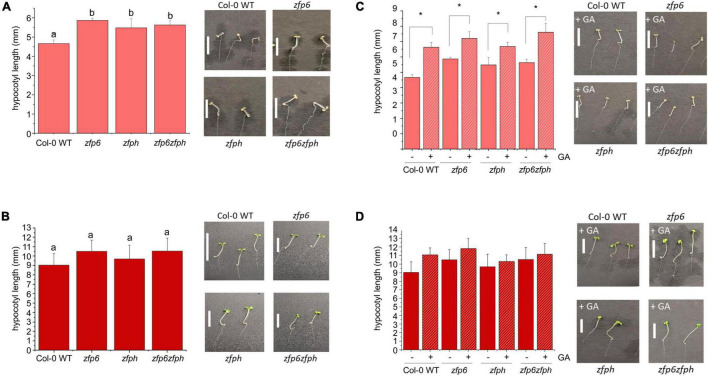
Hypocotyl measurements in FR and R light-exposed seedlings. Seedlings were stratified at 4°C for 4 days in dark on MS plates with 1% sucrose, and subsequently incubated in continuous far-red FR light or R light for 7 days at 22°C. **(A,B)** Untreated seedlings on MS plates exposed to **(A)** FR or **(B)** R light. **(C,D)** Seedlings on MS plates with (+) or without (–) the addition of 10 μM GA under **(C)** FR or **(D)** R light. Hypocotyl measurements were performed using Photoshop 2021 with 3 plates per treatment, each containing 10 seedlings (*n* = 30). Significant differences are highlighted with different letters **(A,B)** or asterisks **(C,D)** at *p* ≤ 0.05. Representative images of seedlings under each condition are shown to the right of bar graphs and the bar in the images equals 1 cm.

Aiming to evaluate the phenotypic impact conferred by *ZFP6* and *ZFPH*, we identified homozygous T-DNA mutants for *ZFP6* (i.e., *zfp6*) and *ZFPH* (i.e., *zfph*). We also created a homozygous *zfp6zfph* double mutant via a genetic cross. Given the regulation of *ZFP6* and *ZFPH* mRNA accumulation by phytochromes and by light for *ZFP6*, we examined seedling photomorphogenic phenotypes in R and FR light grown seedlings. In FR light, the *zfp6, zfph*, and *zfp6zfph* mutant lines all exhibited significantly longer hypocotyls (∼1.2-fold longer) than Col-0 WT (Figure 3A). Although mutant seedlings trended longer that WT under R light conditions, hypocotyl elongation was not significantly different in R conditions (Figure 3B). Noted differences in hypocotyl elongation were light-specific as there was no difference among WT, *zfp6, zfph*, and *zfp6zfph* for seedlings grown in darkness (Supplementary Figure 1).

In addition to the impact of light, seedling growth is tightly regulated by plant hormones. For instance, auxins and GA promote plant growth while abscisic acid is generally known as a plant-growth inhibitor. Given the importance of GA in promoting elongation in seedlings and the prior report of GA regulation of *ZFP6* (Zhou et al., 2013), we evaluated the effect of GA or inhibition of GA accumulation using the pharmacological agent paclobutrazol (PAC) on *zfp6, zfph*, and *zfp6zfph* mutant lines. Seedling hypocotyl length was significantly increased by ∼1.3-fold on average in *zfp6, zfph*, and *zfp6zfph* seedlings treated with GA compared to their corresponding untreated seedlings in FR, which was slightly less than the 1.4-fold longer seedlings observed for GA-treated WT seedlings (Figure 3C). Adding the GA biosynthesis inhibitor PAC disrupted the germination process in all seedlings exposed to FR light, including Col-0 WT. To overcome this, seeds were stratified on MS media without PAC and grown under W light for ∼2 d to allow germination, after which, they were exposed to PAC under FR conditions. All FR-grown, PAC-treated seedlings exhibited significantly shorter hypocotyls than their untreated counterparts, with no significant differences observed between WT and mutants (Supplementary Figure 2A). For R light-grown seedlings, we observed a moderate increase in hypocotyl lengths for GA-treated seedlings compared to control conditions for all lines including WT (Figure 3D), although these differences were not statistically significant as they were under FR. R-light, PAC-treated seedlings exhibited significantly shorter hypocotyls than untreated seedlings for all lines tested inclusive of WT (Supplementary Figure 2B). We also assessed hypocotyl lengths of seedlings under W light, where there were no significant changes in hypocotyl length observed among Col-0 WT and the *zfp6* mutants (Supplementary Figure 3A). There were also no significant changes in root lengths for any of the seedlings lines grown in R, FR, or W light (Supplementary Figures 3B–D).

### *ZFP6* Overexpression Is Sufficient to Inhibit Hypocotyl Elongation in R and Far-Red Light-Grown Seedlings

**FIGURE 4:**
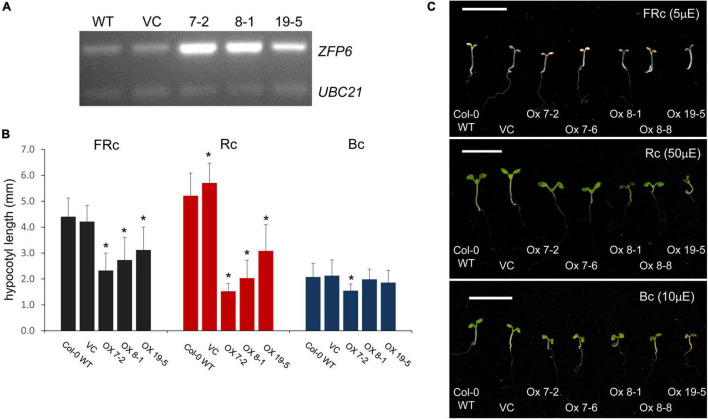
*ZFP6* Overexpression and analyses of hypocotyl lengths of seedlings under different light conditions. **(A)** RT-PCR of *ZFP6* mRNA levels relative to control gene *UBC21* for wild-type, control lines transformed with an empty vector or vector control (VC), and three independent *ZFP6* overexpression lines. **(B,C)** Seedlings were stratified at 4°C for 4 days in the dark, grown in MS plates with 1% sucrose, 0.7% agar, and subsequently incubated in different light conditions for 7 days at 22°C. Seedlings (Col-0 WT, VC lines, and different *ZFP6* overexpression (OX) lines (i.e., OX 7-2, OX 7-6, OX 8-1, OX 8-8, and OX 19-5) were grown under continuous far-red (FRc; 5 μmol m^–2^ s^–1^ or μE), red (Rc; 50 μmol m^–2^ s^–1^ or μE), and blue (Bc; 10 μmol m^–2^ s^–1^ or μE) light. **(B)** Hypocotyl measurements were performed using Image J with at least 25 seedlings per line. Significant differences are shown with asterisks at *p* ≤ 0.05. **(C)** Representative images of seedlings are shown. Bar, 1 cm.

An absence of *ZFP6* and *ZFPH* expression resulted in longer hypocotyls in FR light compared to WT; hence, we hypothesized that overexpression of *ZFP6* or *ZFPH* may inversely result in shorter hypocotyls in seedlings. As expected, elongated hypocotyls observed in *zfp6* seedlings were inversely shortened in *ZFP6*-overexpression (OX) lines (Figure 4). In multiple transgenic lines exhibiting elevated levels of *ZFP6* mRNA (Figure 4A), the inhibition of hypocotyl elongation was impacted compared to WT and vector control (VC) lines (Figures 4B,C), particularly under R and FR conditions. The strongest reduction occurred under R light conditions for the homozygous *ZFP6* #7-2 OX line, compared to Col-0 WT and VC seedlings. Hypocotyl elongation phenotypes were not affected in *ZFP6* OX seedlings treated with blue light, with the exception of a reduction observed for *ZFP6* #7-2 OX in blue light, suggesting a direct interaction between phys and *ZFP6* in the regulation of hypocotyl length. There were no differences for any lines grown in darkness.

**FIGURE 5:**
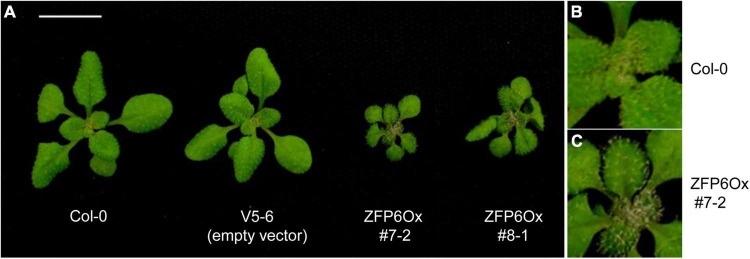
Phenotypes of *35S:ZFP6* overexpression (OX) transgenic lines. **(A)** Rosette structure of Arabidopsis Col-0 WT, vector control (empty vector, i.e., V5-6), and the *35S:ZFP6* OX transgenic lines 7-2 and 8-1. Trichome formation in **(B)** Col-0 WT and **(C)** *ZFP6* OX 7-2 line.

Besides the marked reductions in the lengths of hypocotyls observed for seedlings overexpressing *ZFP6*, we also noted other phenotypic changes for *ZFP6* OX plants. Mature 21 day-old *ZFP6* OX plants grown on soil at 22°C under long days (16 h light, 8 h dark) developed smaller rosettes than Col-0 WT or empty vector plants (Figure 5). Moreover, *ZFP6* OX lines developed more trichomes on their rosette leaves than Col-0 WT plants, which is consistent with a previous report (Zhou et al., 2013).

### Light- and Growth-Responsive Genes Are Differentially Regulated in *ZFP6*- and *ZFPH*-Deficient Lines

**FIGURE 6:**
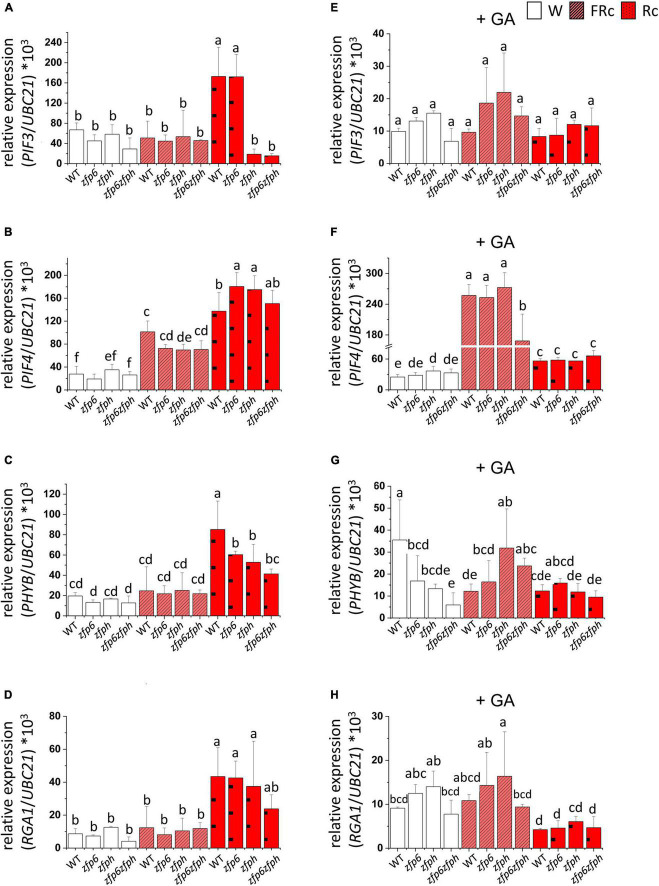
*PIF3, PIF4, PHYB*, and *RGA1* expression in seedlings grown in white, far-red, and red light conditions with or without GA. Seedlings were stratified at 4°C for 4 days in darkness and then incubated for 7 days at 22°C in white (W; 110 μmol m^–2^ s^–1^), continuous far-red (FRc; λmax ∼735 nm at 5 μmol m^–2^ s^–1^), or continuous red (Rc; λmax ∼670 nm at a fluence ∼25 μmol m^–2^ s^–1^) in the absence **(A–D)** or presence of GA (+ GA; **E–H**). *UBC21* was used as the reference gene and the expression data was calculated using the 2^–Δ*CT*^ method. Bars with different letters are significantly different.

Using qRT-PCR, we evaluated the expression of *PIF3, PIF4, PHYB*, and *RGA1* (*Repressor of GA1*), key genes participating in the light- or hormone-dependent regulation of tissue growth (Figure 6; de Lucas et al., 2008; Leivar et al., 2008; Oh et al., 2014). RGA1 is one member of the DELLA family of proteins that binds to PIF3 and PIF4, inhibiting DNA binding activity of these PIF proteins and thus affecting the expression of PIF3- and PIF4-regulated genes (de Lucas et al., 2008; Feng et al., 2008). In R light, target gene *PIF3* was ∼2.6-fold upregulated in Col-0 WT compared to W light-treated Col-0 WT (Figure 6A). Notably, *PIF3* mRNA levels were significantly reduced in the *zfph* and *zfp6zfph* mutants (Figure 6A). This result indicated a positive role for ZFPH in R-dependent *PIF3* mRNA accumulation.

In FR and R light conditions, *PIF4* mRNA levels significantly increased in Col-0 WT by ∼3.7- and ∼5-fold, respectively, compared to W light (Figure 6B). These findings align with a previous report where *PIF4* mRNA levels in Arabidopsis seedlings grown for 6 days in long days (16 h light, 8 h dark) increased ∼1.7–fold after the seedlings were incubated in continuous R light (Zhai et al., 2020), and prior reported upregulation of *PIF4* in both R and FR (Huq and Quail, 2002). Under FR light, *PIF4* expression was ∼1.5-fold reduced in *zfp6* homolog mutant lines compared to Col-0 WT, although the difference was only significant for *zfph*. In *zfp6* and *zfph* single mutant lines treated with R light, *PIF4* transcripts were significantly upregulated by ∼1.3-fold compared to mRNA levels for R light-treated Col-0 WT seedlings.

*PHYB* expression levels were significantly higher in R light-treated seedlings than for W or FR light-exposed plants by an average of ∼3.1-fold (Figure 6C). It has been documented that *PHY* genes are generally constitutively expressed under different light conditions (Clack et al., 1994); however, another report suggests that *PHYB* is transcriptionally regulated (Somers and Quail, 1995). In R light, single and double *zfp6* homolog mutants showed lower *PHYB* expression levels (∼1.7-fold reduction) than Col-0-WT, suggesting that ZFP6 is involved in upregulating *PHYB*.

*RGA1* mRNA levels significantly increased in all R light-treated seedlings by ∼4.4-fold in comparison to W and FR light, with the exception of the *zfp6zfph* double mutant that had an increase but it was only marginally significant. However, no significant differences were detected among R light-treated Col-0 WT and R light-treated *zfp6* mutant lines (Figure 6D).

Given that some *ZFP* genes exhibit cascade or reciprocal regulation and to facilitate interpretation of results for target genes, we tested whether *ZFPH* expression was impacted in a *zfp6* mutant, as well as whether *ZFP6* expression was impacted in the *zfph* mutant background. *ZFP6* does not directly control *ZFPH* expression as *ZFPH* transcripts were present at near WT levels in *zfp6* lines (Supplementary Figure 4). Likewise, *ZFPH* does not act upstream to impact *ZFP6* as *ZFP6* transcripts were present at near WT levels in *zfph* lines (Supplementary Figure 4).

### Gibberellic Acid Modulates Light- Dependent mRNA Levels of Light- and Growth-Responsive Genes in *ZFP6* and *ZFPH* Deficient Lines

Given the prior association of GA with an induction of *ZFP6* expression and the noted impact of light and phytochromes on *ZFP6* and *ZFPH*, we examined the impact of GA on the light- and growth-responsive genes assessed in WT, *zfp6, zfph*, and *zfp6zfph* lines. The addition of GA to the growth media resulted in a modulation of *PIF3, PIF4, PHYB*, and *RGA1* expression levels in a light-dependent manner (Figure 6). The mRNA levels of *PIF3* were not different among lines grown in the presence of GA; yet, this result in the presence of GA represents a loss of R light-associated induction of *PIF3* mRNA levels in WT and *zfp6* lines compared to growth in R light in the absence of GA (Figure 6A vs. Figure 6E). Thus, the R-induced accumulation of *PIF3* appears to be dependent on both GA and ZFPH.

The mRNA levels of *PIF4* were significantly upregulated in FR light-exposed seedlings compared to seedlings grown in R and W light in the presence of GA by ∼7.7- and 4-fold, respectively (Figure 6F). Notably, the pattern of *PIF4* transcript levels in FR and R light-treated seedlings were inverted when the seedlings grew in the presence of GA (Figure 6B vs. Figure 6F). *PIF4* mRNA levels were only significantly different in the *zfp6zfph* double mutant for GA-treated seedlings in FR light, suggesting a redundant role for the two factors under this condition.

By comparison, *PHYB* transcript levels were significantly lower in GA-treated *zfp6, zfph*, and *zfp6zfph* mutant seedlings under W light compared to WT (Figure 6G). While there were no significant differences in *PHYB* mRNA levels for any seedlings including WT when treated with GA under R light, *PHYB* levels were significantly reduced (∼4.8-fold) in GA-treated, R light-exposed seedlings compared to their untreated counterparts grown in R light (Figure 6C vs. Figure 6G). In FR light, *PHYB* was ∼2.6-fold upregulated in GA-treated *zfph* seedlings compared to GA-treated FR light-grown Col-0 WT, and also significantly upregulated in the *zfp6zph* double mutant (Figure 6G).

There was no significant impact of GA treatment on *RGA* mRNA levels in W or FR light conditions (Figure 6H). However, *RGA1* mRNA levels were significantly reduced (∼7.5-fold) in GA-treated R-light exposed seedlings compared to R light-exposed seedlings grown without the addition of GA (Figure 6D vs. Figure 6H).

Together, these results indicate interactions between light, GA and, ZFP6/ZFPH in regulating the expression of some genes, including *PIF3* and *RGA1*.

### *ZFP6* and *ZFPH* Promoters Contain Light-Responsive Elements and Are Potentially Regulated by Light-Induced Genes

**FIGURE 7:**
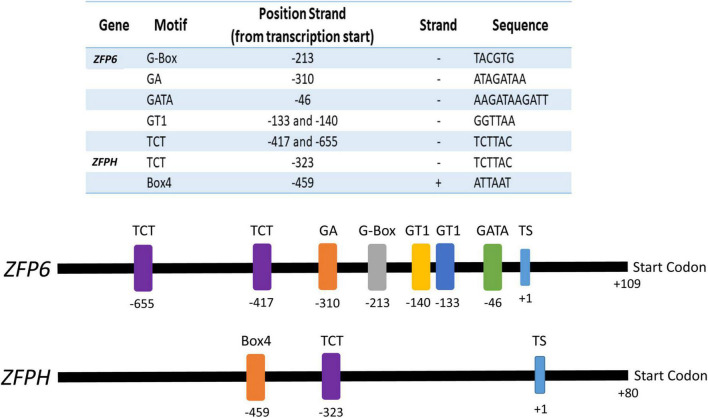
Promoter analyses for *ZFP6* and *ZFPH* genes. The transcription start (TS) was identified using the neural network promoter prediction website and the putative light-responsive promoter regions were found using the PlantCare database (see text for details). Numbers indicate the motif and start codon positions. Sequence of the motif and the strand of DNA on which it is found (–, negative; +, positive) is indicated in the table.

Analyses of the *ZFP6* and *ZFPH* promoter regions were conducted to identify cis-elements potentially involved in light responsiveness. Potential transcription start (TS) sites for *ZFP6* and *ZFPH* were found at nucleotide –109 (score cutoff 0.98) and –80 (score cutoff 1.0), respectively, from the start codon. *ZFP6* and *ZFPH* promoter region analyses resulted in the identification of different light-responsive motifs (Figure 7). For *ZFP6*, elements identified included G-Box, GA, GATA, GT1, and TCT motifs. The *ZFPH* promoter possessed two consensus sequences belonging to the TCT and Box 4 motifs. All these motifs have been previously documented as light-responsive elements (Shariatipour and Heidari, 2018).

To determine whether *ZFP6* and *ZFPH* genes are potentially regulated by proteins encoded by light-responsive genes, an additional *in silico* analysis was performed using the TF2Network database (see text footnote 4). We compared the *ZFP6* and *ZFPH* genes vs. 3,290 genes previously reported as light-responsive genes (**Table 1**; Bechtold et al., 2008; Shi et al., 2018). We found 9 and 17 genes that may encode proteins that bind and potentially regulate expression of *ZFP6* and *ZFPH*, respectively. One notable factor predicted to regulate ZFPH is PIF4, which together with altered *PIF4* levels in the *zfph* mutant in FR and R light conditions (Figure 6A) suggests an interesting potential feedback loop between ZFPH and PIF4. Many of the identified genes belong to the *ZFP* family, which indicates a cascade regulation among *ZFP* genes, as previously reported (Zhou et al., 2013). In addition, among genes predicted to encode factors that regulate *ZFP6* and *ZFPH* are an overrepresentation of hormone-inducible genes, mainly those regulated by ABA (Supplementary Figure 2).

## Discussion

Phytochromes negatively regulate *ZFP6* and *ZFPH* expression. We, thus, investigated whether phytochrome-dependent regulation of *ZFP6* and *ZFPH* is involved in controlling aspects of photomorphogenesis. To examine the interplay between phytochromes and *ZFP6* and *ZFPH* during development, we analyzed the development of *zfp6, zfph* and *zfp6zfph* mutants under distinct light conditions. Given the prior association of *ZFP6* induction by GA, we also examined the impact of modulating GA levels on development through treatment of seedlings with exogenous GA or a GA inhibitor. The *zfp6, zfph*, and *zfp6zfph* mutant lines exhibited significantly longer hypocotyls than Col-0 WT under FR light conditions. There was no specific effect of GA treatment or inhibition of GA accumulation on *zfp6, zfph*, or *zfp6zfph* seedling relative to WT in either R or FR light, indicating that the impacts of GA and phytochromes on these *ZFP* homologs may occur independently.

In FR light, PIFs escape phyB-mediated degradation as phyB remains in the cytosol and thus its transcriptional activity is blocked (Kevei et al., 2007); this FR-associated block of phyB translocation and a lack of associated phyB activity such as the downregulation of *PIF4* in the nucleus promotes PIF4 accumulation and elongated hypocotyls (Fiorucci and Fankhauser, 2017). In line with this, we observed that *PIF4* transcripts significantly increased in response to FR light in Col-0 WT (Figure 6B). As previously reported, *PIF4* mRNA levels also increase in R light (Figure 6A; Zhai et al., 2020). Of note, the phytochrome-dependent regulation of a transcription factor that results in downregulation of *PIF4* mRNA levels in FR light and upregulation in R light in deficient mutants was previously reported for *sig2* mutants (Oh and Montgomery, 2013), which parallels the response noted here for *zfp6* and *zfph* mutants. Of note, SIG2 is a regulatory factor also controlled by phyA and phyB and that impacts both *PIF4* mRNA levels and hypocotyl elongation among other phenotypes (Oh and Montgomery, 2013). However, the regulation of *PIF4* levels did not correspond with significantly longer hypocotyls in R or FR light for *zfp6* and *zfph* mutants. Thus, although ZFP6 and ZFPH appear to exert positive transcriptional regulation on *PIF4* under FR light and negative regulation under R light, this does not explain in full the significant disruption in hypocotyl elongation under FR. This finding may suggest that other members of the PIF family, or other factors altogether, may be involved in coordinating the observed etiolated responses in FR light where the hypocotyls of *ZFP6* and *ZFPH*-deficient seedlings were significantly longer than WT. We also checked *PIF3* mRNA levels in FR and its expression was not significantly changed under these conditions.

**FIGURE 8:**
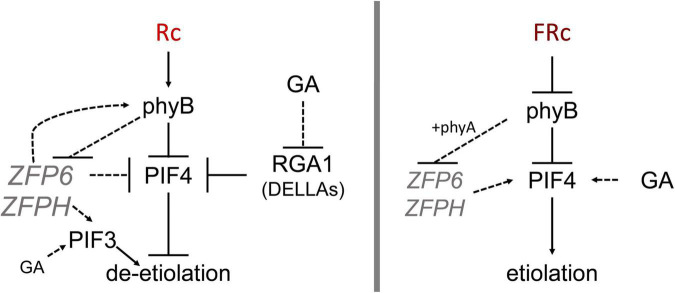
Model showing ZFP6 genetic interactions in red and far-red light. Published literature has demonstrated that phyB destabilizes PIFs while RGA1 (a DELLA family protein) blocks PIF4 (prior relationships represented with solid black lines). In red (R) light (left), phyB reduces *ZFP6* expression. In turn, ZFP6 downregulates *PIF4* and induces *PHYB* (relationships established in this work represented with dotted lines) in R light likely providing a feedback loop to aptly modulate hypocotyl lengths in response to light. ZFPH serves to promote *PIF3* in R light, and *PIF3* is also promoted by GA in R. In the presence of the growth-promoting GA hormone in R light, GA reduces *RGA1* transcripts, which allows PIF4 accumulation and promotion of elongation in presence of GA. In far-red (FR) light (right), phyB together with phyA reduce *ZFP6* (and *ZFPH*) expression. In the presence of the growth-promoting GA hormone in FR, GA promotes *PIF4* accumulation leading to elongation typical of etiolation.

Under R light, our results imply that ZFP6 may limit hypocotyl elongation in part in WT by blocking *PIF4* mRNA accumulation in R light (Figure 8), taking into consideration previous research that has shown consistency at the level of transcript levels and protein accumulation for PIF (Lee et al., 2021) and DELLAs (Zentella et al., 2007; Achard et al., 2008). To demonstrate whether *ZFP6* is sufficient to inhibit hypocotyl elongation in seedlings, we created transgenic plants overexpressing *ZFP6*. The *ZFP6* OX plants exhibited shorter hypocotyls than Col-0 WT Arabidopsis seedlings, especially those exposed to FR and R light conditions (Figure 4). These results confirm a key regulatory role of *ZFP6* in restraining tissue elongation.

We observed elongated hypocotyls in all cases when GA was added. Additionally, Col-0 WT and all mutant seedlings treated with PAC displayed the same phenotypes independent of whether grown in R or FR light. These results indicate that DELLAs exert their impact on seedling elongation via an independent mechanism compared to *ZFP6* and *ZFPH*, and that DELLAs likely serve as master regulators in response to GA.

As we observed increased *ZFP6* mRNA levels for *phyB* mutants, we were also interested in evaluating the expression of *PHYB* in *zfp6* mutant lines to test for reciprocal regulation. *PHYB* was downregulated in *zfp6* and *zfph* mutant lines grown in R light, suggesting that ZFP6 is implicated in upregulating *PHYB* under these conditions (Figure 8). Indeed, by performing *in silico* analysis of the *ZFP6* and *ZFPH* promoters, we identified several light-regulated motifs in the *ZFP6* and *ZFPH* promoters. We also identified several proteins encoded by light-regulated genes that can potentially regulate *ZFP6* and *ZFPH*. This finding aligns with prior analyses in which some members of the ZFP family have been previously associated with photomorphogenesis in plants (Ito et al., 2018).

Here, we report that hypocotyl elongation can be modulated at the seedling stage depending on *ZFP6* and *ZFPH* phytochrome-dependent regulation. In addition to *ZFP6* and *ZFPH* being regulated by light and phytochrome activity, ZFP6 and ZFPH regulate *PHYB* and *PIF4* and *PIF3*, key components of the photomorphogenesis signaling cascade that can impact organ elongation genes. In mature plants, the rosette architecture is markedly reduced in lines overexpressing *ZFP6*, while the hairy trichomes become denser as previously reported (Zhou et al., 2013). Additional research is needed to fully elucidate the phytochrome and *ZFP6/ZFPH*-dependent regulatory network(s) that target organ-elongation genes and, ultimately, control light-dependent morphogenesis *in planta*.

## Data Availability Statement

The original contributions presented in the study are included in the article/Supplementary Material, further inquiries can be directed to the corresponding author/s.

## Author Contributions

KC-R and SO designed and conducted the research, analyzed and interpreted data, and contributed to writing and editing the article. BM designed the research, analyzed and interpreted the data, and contributed to writing and editing article. All authors approved the submitted article.

## Conflict of Interest

The authors declare that the research was conducted in the absence of any commercial or financial relationships that could be construed as a potential conflict of interest.

## Publisher’s Note

All claims expressed in this article are solely those of the authors and do not necessarily represent those of their affiliated organizations, or those of the publisher, the editors and the reviewers. Any product that may be evaluated in this article, or claim that may be made by its manufacturer, is not guaranteed or endorsed by the publisher.

## Footnotes

1
https://abrc.osu.edu/

2
http://bioinformatics.psb.ugent.be/webtools/plantcare/html/

3
https://www.fruitfly.org/seq_tools/promoter.html

4
http://bioinformatics.psb.ugent.be/webtools/TF2Network/

5
https://www.arabidopsis.org/portals/expression/microarray/ATGenExpress.jsp
