# Supplementary material for: Phytochrome-Dependent Regulation of ZFP6 and ZFPH Impacts Photomorphogenesis in Arabidopsis thaliana
Source: Front Plant Sci. 2022 Jun 1;13:846262. doi: 10.3389/fpls.2022.846262 (PMC9198550; doi:10.3389/fpls.2022.846262)

Supplementary Material

Phytochrome-dependent regulation of *ZFP6* and *ZFPH* impacts photomorphogenesis in *Arabidopsis thaliana*

Keni Cota-Ruiz[1], Sookyung Oh[1], Beronda L. Montgomery[1,2,3]

[1]MSU DOE-Plant Research Laboratory, Michigan State University, East Lansing, MI 48824, USA

[2] Department of Biochemistry and Molecular Biology, Michigan State University, East Lansing, MI 48824, USA

[3]Department of Microbiology & Molecular Genetics, Michigan State University, East Lansing, MI 48824, USA

*** Correspondence:**
Corresponding Author
montg133@msu.edu

Keywords: phytochrome, *ZFP6*, *ZFPH*, gibberellic acid, PIF, DELLA, far-red light

Supplemental Table S1. **List of Primers and RT-PCR conditions**

Gene	Forward sequence	Reverse sequence	Cycling conditions
ZFP6 (RT-PCR)	ACGTCGGTTCAA AACCAGTC	TCCAGCCCAAT ACCATTCTC	95 °C for 3 min; 27 cycles at 94 °C for 30 s, 58 °C for 30 s, and 72 °C for 30 s; 72 °C for 5 min
ZFP6 (qRT-PCR)	GAAGGGGACTTT ACGGAGGTG	GGTCCAGCCCA ATACCATTCT	95 °C for 5 min; 40 cycles at 95 °C for 15 s, 61 °C for 25 s, and 72 °C for 20 s
ZFPH (qRT-PCR)	TCATCCAAGATG GTTCGGGTG	TGAGCCCCAAC TACTCCAAAC	95 °C for 5 min; 40 cycles at 95 °C for 15 s, 61 °C for 25 s, and 72 °C for 20 s
PIF3 (qRT-PCR)	ACGATTTGGTCCC CTCTCTT	AAAGTCGGCGA ATTGGAGTA	95 °C for 5 min; 40 cycles at 95 °C for 15 s, 56 °C for 25 s, and 72 °C for 20 s
PIF4 (qRT-PCR)	TCAGATGCAGCC GATGGAGATG	CAGACGACGGT TGTTGACTTTGC	95 °C for 5 min; 40 cycles at 95 °C 15 s, 60 °C 25 s, and 72 °C 20 s
PHYB (qRT-PCR)	GCTGTGACCAGTC AAGATCCA	CACCTGTCACTA CACCTTCCC	95 °C for 5 min; 40 cycles at 95 °C for 15 s, 57 °C for 25 s, and 72 °C for 20 s
RGA1 (qRT-PCR)	GGTACGCAGATT GGTGGAGT	AACACCGTTCTC TTGCGAGT	96 °C for 5 min; 40 cycles at 95 °C for 15 s, 57 °C for 25 s, and 72 °C for 20 s
UBC21 (RT-PCR)	CCTTACGAAGGC GGTGTTTTTCAG	CGGCGAGGCGT GTATACATTTG	95 °C for 3 min; 27 cycles at 94 °C for 30 s, 58 °C for 30 s, and 72 °C for 30 s; 72 °C for 5 min
UBC21 (qRT-PCR)	CAAATGGACCGC TCTTATCAAAG	CTGAAAAACAC CGCCTTCGT	95 °C for 5 min; 40 cycles at 95 °C for 15 s, 61 °C for 25 s, and 72 °C for 20 s

Supplemental Table S2. Analysis of genes that codify for proteins that may bind to *ZFP6* and *ZFPH* genes

See Excel file.

Supplemental Figure S1. **Hypocotyl lengths and representative image for dark-grown seedlings**. Seeds were stratified at 4°C for four days in dark, then grown in MS plates with 1% sucrose, and incubated in continuous darkness for seven days at 22°C. (A) Hypocotyl measurements were performed using Image J with at least 25 seedlings per line. (B) Representative images of seedlings are shown.

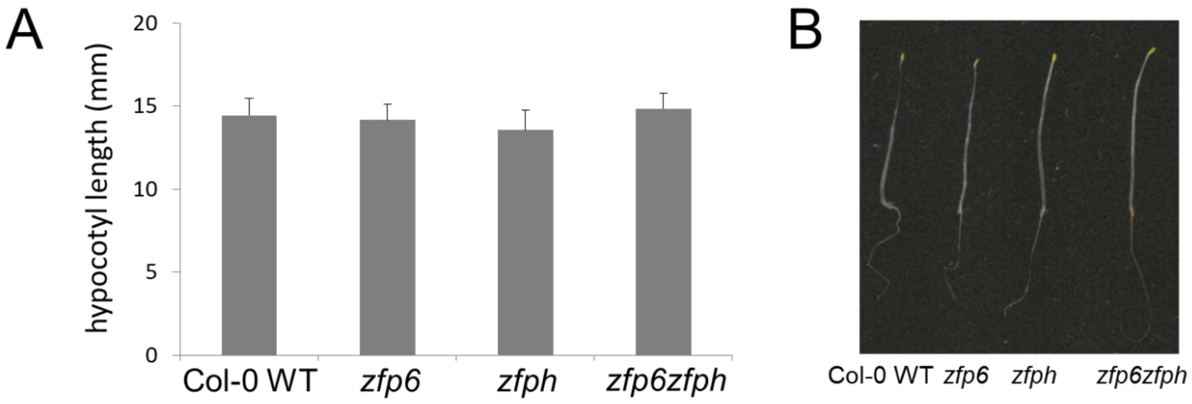

Supplemental Figure S2. **Hypocotyl lengths in far-red and red light-treated seedlings with and without GA inhibitor PAC**. Seeds were stratified at 4°C for four days in dark, then grown in MS plates with 1% sucrose without (-) or containing (+) 100 nM paclobutrazol (PAC), and incubated in continuous (A) far-red [FR] or (B) red [R] light for seven days at 22°C. Hypocotyl measurements were performed using Image J with at least 25 seedlings per line. Significant differences are shown with asterisks at $p \leq 0.05$.

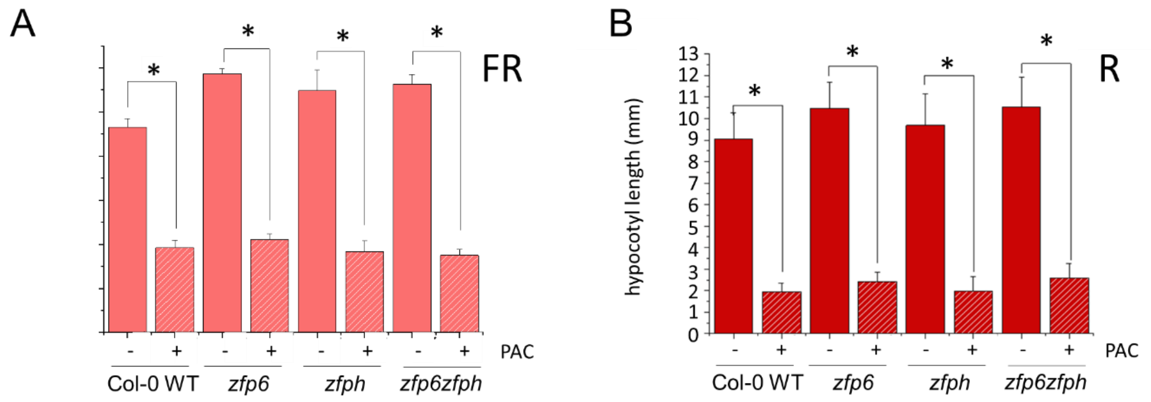

Supplemental Figure S3. **Hypocotyl and root measurements of seedlings grown in different light conditions**. Seeds were stratified at 4°C for four days in dark, then grown in MS plates with 1% sucrose for seven days at 22°C, and incubated in (A) white (W) light for hypocotyl length measurements, (B) W for root length measurements, (C) continuous red (Rc) for root length measurements, and (D) continuous far-red (FRc) light for root length measurements.

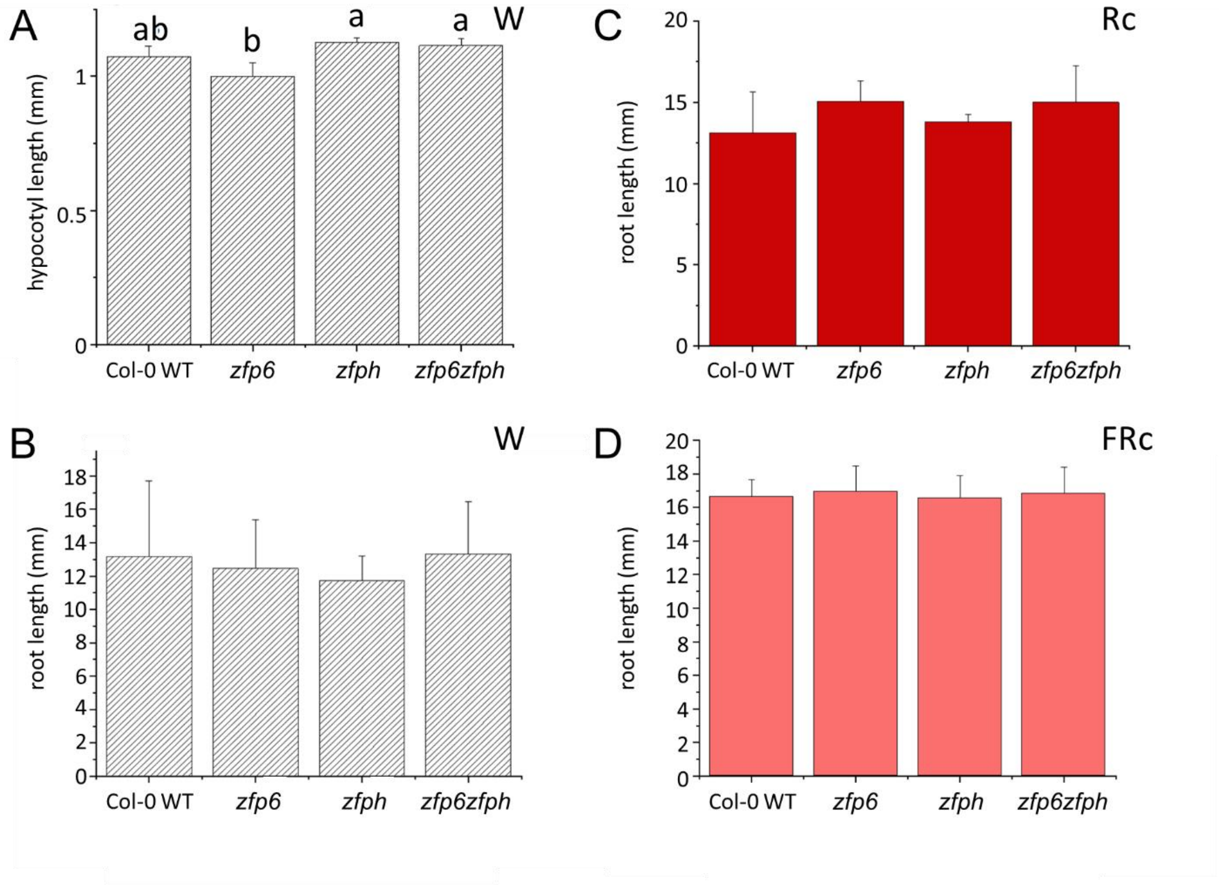

Supplemental Figure S4 . *ZFP6* and *ZFPH* expression in Col-0 WT, *zfp6*, *zfph* and *zfp6zfph* lines. (A) *ZFP6* and (B) *ZFPH* transcript levels were quantified via qRT-PCR from 100 ng total RNA using RNA extracted from Col-0 WT, *zfp6*, *zfph* and *zfp6zfph*. Bars represent the mean of three biological replicates ± SD.

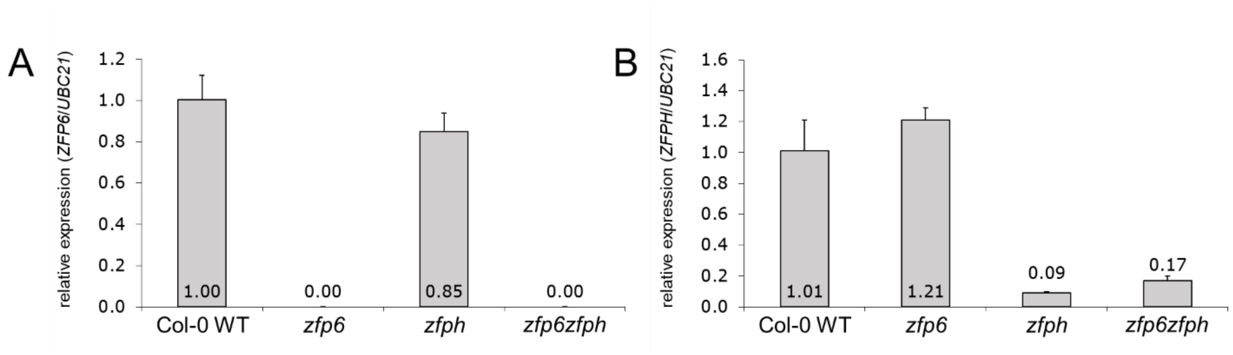

Supplement: Supplementary file 1 [file Data_Sheet_1.pdf]
